# Transfer RNA regulation during herpesvirus infection: Wielding a sword or raising a shield?

**DOI:** 10.1371/journal.ppat.1013750

**Published:** 2025-12-05

**Authors:** Kyle Rapchak, Jessica M. Tucker

**Affiliations:** 1 Department of Microbiology and Immunology, Carver College of Medicine, University of Iowa, Iowa City, Iowa, United States of America; University of Iowa, UNITED STATES OF AMERICA

## Introduction

Infection represents a continuous battle between the host and the pathogen, each striving to control the cellular environment and gain an advantage. Viruses are adept at hijacking and antagonizing host machinery to promote their own replication and spread. Once inside a host cell, they use host machinery to build and release infectious particles, commandeer cellular pathways, block the interferon response, and promote host cellular proliferation [1–3]. In defense against infection, host cells activate a range of innate immune responses to deplete biomolecules, alter surface protein expression, or destroy viral replication sites [4–7]. Central to this conflict is the regulation of gene expression; ultimately, the ability to control gene expression can determine whether the host or the virus gains the upper hand.

**Fig 1:**
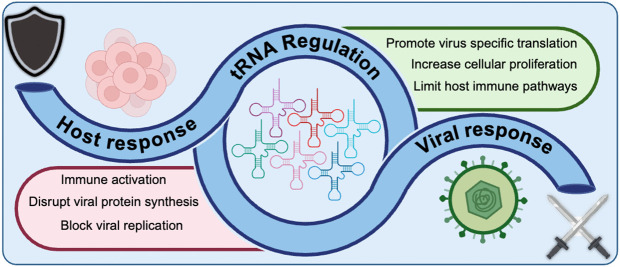
Graphical summary: Transfer RNA regulation offers offensive and defensive strategies during viral infection. Created in Biorender.

Research on gene expression regulation during infection has traditionally centered on messenger RNA (mRNA) dynamics, including transcription by RNA polymerase II and translation by the ribosome [8–10]. However, non-coding RNAs also significantly influence infection outcomes. Among these, transfer RNAs (tRNAs)—transcribed by RNA polymerase III—serve as essential adapters in translation and are the focus of this discussion. By facilitating protein synthesis, tRNAs play a dual role in supporting the host’s innate immune response and enabling the production of viral proteins required for replication. There is mounting evidence that herpesviruses dramatically increase tRNA expression during lytic infection, with representatives from all three subfamilies (*Alpha-*, *Beta-*, and *Gammaherpesvirinae*) inducing this response. Upregulation of tRNA expression occurs through multiple mechanisms, including an increase in RNA polymerase III gene occupancy, RNA polymerase II/III crosstalk, and alteration of chromosome around tRNA genes [11–13]. The strength and representation of this phenotype among distinct herpesviruses suggests that tRNA production plays a fundamental role during infection. Nevertheless, it remains to be experimentally demonstrated whether tRNA upregulation benefits the virus or serves as a host response to infection. This review aims to explore how tRNA function could be linked to herpesvirus infection and considers scenarios in which tRNA induction may contribute to either viral propagation or host resistance (Fig 1). A deeper understanding of this relationship could reveal new strategies for manipulating the tRNA landscape to influence infection outcomes.

## What do we know about tRNA expression and regulation?

Discovered over 60 years ago, tRNAs were the earliest identified non-coding RNAs, fundamentally transforming our understanding of RNA function in biological systems [14]. tRNAs are small RNAs (76–90 bases) initially synthesized by RNA polymerase III as unmodified precursors, known as premature tRNAs [15]. These precursors undergo a series of processing steps and base modifications, followed by aminoacylation, resulting in mature tRNAs charged with their corresponding amino acids. The canonical role of tRNAs is to serve as adapters within the ribosome, facilitating protein synthesis [16]. During translation, aminoacylated tRNAs recognize and bind to specific codons through their anticodon loops, allowing the ribosome to assemble peptide chains. The genetic code includes 61 different codons that encode 20 different amino acids. This redundancy results in groups of tRNAs—called isoacceptors—with different anticodon sequences that can be charged with the same amino acid [17]. Additionally, tRNA genes are highly duplicated in the human genome, with approximately 400 tRNA genes predicted to be functional. It is hypothesized that the high copy number and redundancy of tRNA genes can benefit cells by supporting robust protein synthesis during translation.

Interestingly, a variable half of all tRNA genes are either selectively silenced or expressed at low levels across different human cell types [18,19]. This might be explained by the observation that synthesis of excess tRNAs consumes valuable cellular resources [20], but the mechanism underlying the selective expression of tRNA genes remains poorly understood. For translation to proceed efficiently, the tRNA pool within a cell must uniquely support decoding of the transcriptome in use [21–23]. Certain gene expression programs (i.e., differentiation or tissue-specific programs) exhibit unique codon biases, meaning that tRNA repertoires vary across tissue type, developmental stage, or environmental condition within the same organism [24–26]. Additionally, viruses often display codon usage frequencies that differ from their host and have evolved strategies to overcome these barriers to ensure viral proteins are made in high abundance [27,28]. Collectively, these observations suggest that mammalian tRNA expression is tightly regulated in response to environmental cues, including viral infection, though the full extent of this regulation remains to be elucidated.

Beyond serving as adapters in translation, tRNAs also perform several non-canonical activities during cellular stress and infection. For example, stress commonly triggers the cleavage of tRNAs by endonucleases, producing tRNA-derived fragments (tRFs) with regulatory activities similar to miRNAs or other small non-coding RNAs [29]. Reported functions of tRNA fragments include translation inhibition, mRNA repression, modulation of the DNA damage response, and tumor suppression [30–34]. tRFs can also act as amino acid donors in specialized biosynthetic processes, including post-translational protein modification [35]. Additionally, tRNAs can bind or sequester proteins to modulate their activity. For example, tRNAs can competitively bind cytochrome C away from Apaf-1, an apoptosome assembly protein, and block its ability to function in apoptotic pathways [36,37]. Other known functions include nutrient sensing via accumulation of uncharged tRNA pools, regulation of immune sensing, and priming of reverse transcription [38–40]. Together, these canonical and non-canonical functions of tRNAs underscore the central role of tRNAs in regulating gene expression during homeostasis and in response to stress and infection, making their regulation relevant in the context of virus-host interactions.

## How are tRNAs regulated during herpesvirus infection?

The *Herpesviridae* family consists of three subfamilies, encompassing a total of nine human-infecting viruses. Of these, three viruses (one from each subfamily) have been reported to alter the tRNA landscape during infection [11,12,41]. As mentioned above, herpesviruses employ diverse mechanisms to modulate tRNA expression. Herpes simplex virus 1 (HSV-1), an alphaherpesvirus, induces tRNA expression through enhanced RNA pol II binding and crosstalk with RNA pol III at tRNA genes [12]. Similarly, human cytomegalovirus (HCMV), a betaherpesvirus, activates tRNA expression in response to infection-induced changes in chromatin state [11]. Epstein-Barr virus (EBV), one of the two human gammaherpesviruses, has also been shown to increase tRNA production by upregulating RNA pol III transcription factors during latency [41]. Our studies using the murine model gammaherpesvirus MHV68 revealed transcriptional upregulation in combination with increased stability and/or reduced turnover of pre-tRNAs that contributes to an overall elevated abundance during lytic infection [13]. Additionally, tRNA fragments made from premature and mature tRNAs are generated in response to MHV68 infection [42]. The biogenesis of tRNA fragments has yet to be demonstrated during other herpesvirus infections. It will be important to dissect if the described mechanisms for tRNA upregulation are virus-specific, or if they reflect a broader, conserved cellular response. Future investigations should also explore whether human herpesviruses induce tRNA fragments (as observed with MHV68 [42]), alter tRNA modification patterns, or affect tRNA charging status, to fully elucidate how tRNAs are regulated during infection.

## Is tRNA regulation beneficial to the host or the virus?

Given the importance of tRNA expression on gene expression control, it is logical to predict that the host and virus employ mechanisms to subvert or counteract mechanisms to regulate tRNA transcription and processing. Though tRNA transcription is upregulated during herpesvirus infection, it is not known which party—host or virus—benefits from these changes, nor the key proteins that drive tRNA regulation in the infected cell. We present two main scenarios driven by tRNA regulation in the cell that require further study:

1 Altered tRNA pools can support host or virus codon preference:

Codon usage, especially when it diverges between host and virus, is a key factor in understanding how tRNA regulation influences host-virus interactions. Across species, from bacteria to humans, tRNA cleavage has emerged as a mechanism to inactivate tRNAs and restrict viral protein synthesis. For example, *Escherichia coli* uses a tRNase called PrrC to nick and disable tRNA^Lys^ during T4 phage infection to block viral translation [43]. Similarly, human cells unleash endonucleases to reduce the abundance of specific tRNAs, particularly those required to decode codons enriched in viral transcripts [44–46]. One such endonuclease is Schlafen 11, an interferon-stimulated protein that cleaves tRNAs during infection and inhibits protein synthesis in response to infection by HCMV, as well as HIV-1, influenza A virus, and various flaviviruses [46–49]. To evade these restrictions, some viruses may adopt codon usage patterns that closely match those of their host, thereby minimizing reliance on rare or targeted tRNAs [50,51]. Conversely, viruses might benefit from reshaping the host tRNA landscape to both align with their codon preference and to promote cellular proliferation and resource acquisition. It has been proposed that certain host pathways—such as those involved in cell cycle progression, DNA repair, and RNA processing—share codon usage profiles with infecting viruses, potentially facilitating viral replication [50]. Regulation of tRNA expression extends to the epitranscriptome, as SARS-CoV-2 and ZIKV infections are associated with differential tRNA modification, specifically ncm^5^U, mcm^5^U, and mcm^5^s^2^U modifications at U34, that appears to not only promote their lifecycle but also negatively impact host transcriptional control [52]. Additionally, viral manipulation of the cell cycle may influence tRNA expression, as proliferating cells possess distinct tRNA profiles compared to differentiated or arrested cells [21,53]. Together, these findings underscore the intricate relationship between codon usage, tRNA regulation, and viral replication strategies—highlighting the need to further investigate how viruses manipulate host tRNA expression, modification, and turnover to optimize their lifecycle and evade immune restriction.

1. Infection-induced tRNA Fragments (tRFs) function as effector RNAs that can support or restrict viral replication:

As mentioned prior, tRNAs are subject to cleavage in response to infection and other forms of cellular stress. In the previous section, we outlined the role of endonucleolytic cleavage to inactivate specific tRNA families and inhibit translation (e.g., [45,54]). However, consequences may extend beyond tRNA inactivation, as many reports indicate that tRNA cleavage does not significantly alter the overall levels of mature tRNAs [55,56]. Instead of tRNA inactivation, some cleavage events produce tRFs that exert effector functions capable of supporting or blocking viral replication [56–59]. The functions of tRFs are remarkably diverse. For example, they can: (1) regulate protein translation and gene silencing in a manner similar to miRNAs—such as RSV-induced tRFs that silence antiviral host transcripts [56,60]; (2) act as immune activators by being secreted and signaling to endosomal Toll-like receptors in neighboring cells; [61] or (3) bind and sequester proteins in a manner than influences infection (i.e. tRF that sequesters proviral factor La/SSB during HCV infection) [57,59]. Accurate profiling and functional studies of tRFs in response to viruses, including the herpesviruses, will be required to dissect the roles of these novel small RNAs employed in the infected cell.

## Conclusion

The mechanisms described above illustrate how tRNA regulation can reprogram gene expression and shape host-pathogen interactions. Investigating these diverse modes of tRNA control—particularly in the context of Herpesviridae, which induce tRNA transcription in a seemingly conserved fashion—offers a promising avenue for discovery across viral systems. A critical first step is to comprehensively define the changes in tRNA and tRF repertoires during infection, which will depend on continued advancements in tRNA and tRF sequencing technologies [62–65]. In parallel, we must refine tools to modulate tRF biogenesis and expression in order to validate their functional roles in viral replication and host defense. Integrating tRNA sequencing with ribosome profiling will be essential for understanding how dynamic shifts in the tRNA/tRF landscape influence translation during infection. Additionally, further exploration of tRFs as RNA-binding protein decoys and other effector molecules will expand our understanding of their mechanistic roles. Ultimately, uncovering the molecular details of tRNA regulation during infection will pave the way for novel therapeutic strategies—including the development of small tRNA-like molecules—to inhibit viral replication and spread, not only in herpesviruses but across a broad spectrum of viral pathogens.
